# Differential Cardiac Effects of Aerobic Interval Training Versus Moderate Continuous Training in a Patient with Schizophrenia: A Case Report

**DOI:** 10.3389/fpsyt.2014.00119

**Published:** 2014-08-29

**Authors:** Marco Herbsleb, Tobias Mühlhaus, Karl-Jürgen Bär

**Affiliations:** ^1^Pain and Autonomic Integrative Research (PAIR), Department of Psychiatry and Psychotherapy, University Hospital Jena, Jena, Germany; ^2^Clinical Exercise Physiology (CEP), Department of Sports Medicine and Health Promotion, Friedrich-Schiller-University, Jena, Germany

**Keywords:** schizophrenia, interval training, continuous training, heart rate variability, exercise intervention

## Abstract

Increased cardiovascular morbidity and mortality rates for patients with schizophrenia are reported to contribute to their reduced life expectancy. Common reasons for increased cardiac mortality rates include cigarette smoking, obesity, dyslipidemia, diabetes, and poorer health behavior in general. The majority of excess mortality among people with schizophrenia is caused by cardiovascular complications. Reduced vagal activity might be one important mechanism leading to this increased cardiac mortality and has been consistently described in patients and their healthy first-degree relatives. In this case study, we compared two different aerobic exercise regimes in one patient with chronic schizophrenia to investigate their effects on cardiovascular regulation. The patient completed a 6-week period of moderate continuous training (CT) followed by a 6-week period of interval training (IT), each regime two times per week, on a stationary bicycle. This was followed by a 6-week period of detraining. Primary outcome measures examined heart rate (HR) and heart rate variability (HRV) at rest while secondary measures assessed fitness parameters such as the ventilatory threshold 1 (VT_1_). We observed that IT was far more effective than moderate CT in increasing HRV, as indicated by root mean of squared successive difference (improvement to baseline 27 versus 18%), and reducing resting HR (−14 versus 0%). Improvement in VT_1_ (21 versus −1%) was only observed after IT. Our study provides preliminary data that the type of intervention is highly influential for improving cardiac function in patients with schizophrenia. While cardiovascular function might be influenced by CT to some degree, no such effect was present in this patient with schizophrenia. In addition, the beneficial effect of IT on HR regulation vanished completely after a very short period of detraining after the intervention.

## Background

Schizophrenia is associated with poor levels of physical health leading to high rates of physical morbidity and mortality (1–3). The relative risk of premature death of patients with schizophrenia is increased by two to fourfold and they are said to die at least 10 years earlier than their age-matched contemporaries (4). A biological predisposition, the illness itself (either through positive, negative, or cognitive symptoms) and an unhealthy lifestyle might contribute to excess mortality from natural causes. Interestingly, poor physical health occurs already at an early age in these patients, and those aged between 25 and 44 years seem to differ most pronouncedly from their age-matched contemporaries. Various studies have suggested increased prevalence and/or mortality rates of some diseases such as diabetes, hypertension, heart disease, gastrointestinal or respiratory disorder, and breast cancer (3–8). However, although risk-factors such as increased weight, smoking rates, poor diet, and activity levels have a critical role in increased risk of morbidity and mortality, they do not account for the entire hazard (9). There seems to be some disease-inherent biological predisposition. The majority of excess mortality among people with schizophrenia is caused by cardiovascular complications, especially coronary heart disease (9). In addition, the prevalence of heart failure or arrhythmia is increased in this population (10). Reduced vagal activity might be one important mechanism leading to this increased cardiac mortality (11) and has been consistently described in patients and their healthy first-degree relatives (12–14). This autonomic imbalance has been investigated by means of heart rate variability (HRV) and complexity studies. High HRV indicates healthy cardiovascular regulation and is suggestive of the ability to adjust to environmental changes. It is associated with long-term survival with respect to various heart conditions. In patients with schizophrenia, parasympathetic parameters of the time domain of HRV such as root mean of squared successive difference (RMSSD) and of the frequency domain such as HF (high frequency) have repeatedly indicated low parasympathetic (vagal) modulation and have been associated with low HRV (15). Similarly, the complexity measure compression entropy (Hc), which reflects vagal modulation to some extent, has been shown to indicate reduced complexity in patients (16, 17). In addition, the relatively novel measure QT variability index (QTvi) indicating cardiac repolarization lability has been studied in the disease and results were suggestive of augmented sympathetic modulation and an increased risk of arrhythmias (18). Interestingly, most studies have reported an association of the autonomic imbalance with the degree of positive symptoms (i.e., delusions) correlating negatively with, e.g., RMSSD or the complexity measure Hc. Moreover, increased HRs have been associated with poor outcomes in schizophrenia, suggesting that heightened SNS activity is an important factor for progression of the disease. Not surprisingly, this abnormal pattern of cardiac regulation is not only present at rest but more importantly during exercise (19). The study by Ostermann et al. (19) showed reduced physical capacity, even after adjusting for weight, and as expected, decreased vagal modulation was associated with psychopathology and a pro-inflammatory response after exercise in patients. Likewise, Heggelund and colleagues showed that male patients suffering from schizophrenia have reduced VO_2peak_ in comparison to the general population. The authors suggest that the assessment of VO_2peak_ should be incorporated into clinical practice for cardiac risk prediction in these patients (20), especially since aerobic high-intensity training has been shown to improve VO_2peak_ in patients with coronary artery disease (21), as well as in patients with schizophrenia (22). Similarly, a number of studies point to a significant improvement of schizophrenia symptoms via aerobic exercise (23, 24). This has also been confirmed by a first meta-analysis (25). However, the increased HR in these patients, which is not primarily associated with neuroleptic medication, has not specifically been addressed by exercise intervention studies.

It is well known that aerobic endurance training induces a reduction in heart rate (HR) at rest and during submaximal exercise intensities (26). Many previous studies have suggested that bradycardia due to exercise may be caused by decreased cardiac sympathetic modulation and increased cardiac parasympathetic modulation (27–30). Therefore, aerobic physical exercise has been proven to be an effective method to decrease HR at rest by increasing its variability (HRV) (31–33). Similarly, data underline this effect during submaximal exercise intensities (34, 35) and after recovery (36). The extent of cardiac effects after aerobic training seems to be related to training intensities and exercise regime (e.g., interval and continuous exercise) (37–39). One study by Helgerud et al. (40) showed that aerobic high-intensity interval training (IT) improved VO_2max_ more than moderate training in healthy college students (40). Interestingly, changes in VO_2max_ corresponded with changes in stroke volume of the heart, indicating a close link between the two measures.

Therefore, it is tempting to speculate that different training regimens might induce different effects on increased HRs and low vagal function in patients with schizophrenia. The purpose of this case study was therefore to explore differential effects on cardiovascular regulation and physical fitness of moderate continuous training (CT) for 6 weeks versus a 6-week period of IT in a female patient suffering from paranoid schizophrenia.

## Case Presentation

A 54-year-old woman came initially to her psychiatrist with delusions of being observed, auditory hallucinations, and severe insomnia when she was 30 years of age. At the time, a diagnosis of paranoid schizophrenia according to ICD-9 criteria was made. First admission to our hospital occurred 8 years ago when she had various delusions, thoughts of references, auditory hallucinations, and overslept regularly. She was treated with risperidone (4 mg/day), and her psychotic symptoms improved substantially. She had lost her job as a cleaning lady during the course of her disease and is married. Her childhood and development were unremarkable. Her medical history revealed a thyroidectomy in 1989 and T3 replacement. No further diseases are known. She experienced menopause at age 50. Her mother’s brother and sister both committed suicide. However, no data are available on whether underlying psychiatric conditions were known. After initial admission, she has been admitted to our hospital about once a year. Positive symptoms decreased over time and negative symptoms became aggravated. During the last admission, she complained mainly about a lack of energy, anhedonia, sleeping problems, and some urinary incontinence. She denied positive symptoms such as hallucinations and reported some delusions of persecution. She complained of an unspecific anxiety, low mood, lack of energy, and worry about the future. She is now treated with ziprasidone (blood level 0.17 μg/ml). She has become obese during the last 5 years (BMI: 40.7) and very mildly incontinent. She has not done any physical exercise for about 20 years. No signs of diabetes, increased glucose levels, heart disease, or any other condition were detectable. Routine ECG and laboratory tests were entirely unremarkable. Her initial ratings on the positive and negative syndrome scale [PANNS; (41)] were: positive scale = 24; negative scale = 42; and general psychopathology 45. She agreed to participate in an exercise intervention and gave written informed consent to a protocol approved by the Ethics Committee of the Medical Faculty of the Friedrich-Schiller-University, Jena.

### Study design

**Figure 1:**
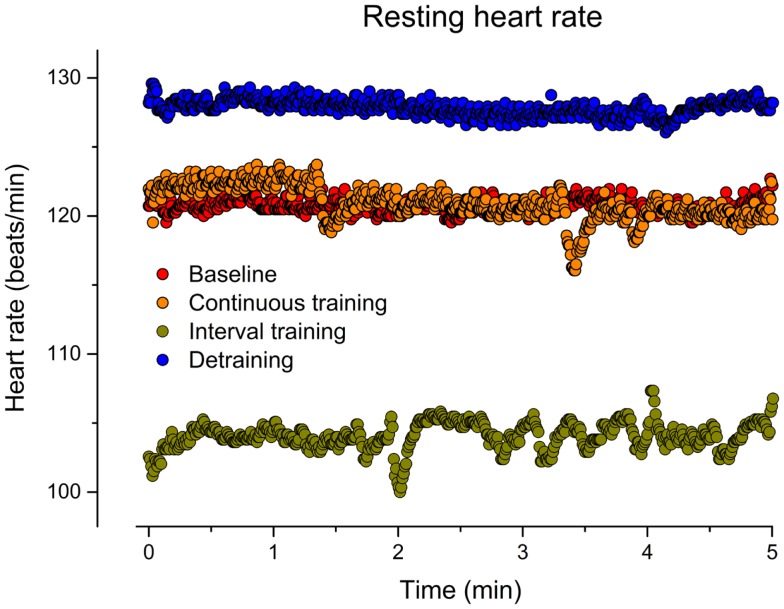
**Resting heart rate (beat-to-beat) before training (baseline), after 6 weeks of continuous training, 6 weeks of interval training, and 6 weeks of detraining**.

The present study involved a total period of 18 weeks, which included a 6-week period of moderate CT followed by a 6-week period of IT and finally 6-weeks of detraining. Before and after each period, we performed a graded cardiopulmonary exercise test (CET) on a stationary bicycle in order to assess her physical fitness and the training effects on cardiac regulation (Figure 1). The patient was instructed to refrain from additional exercise outside of the study requirements as well as from alcohol and caffeine intake for at least 24 h prior to any of the test sessions.

### Baseline and outcome measurements

Three days in advance, and after each training period, the patient performed a CET on an electronically braked cycle ergometer (Ergometrics 900, Ergoline, Bitz, Germany). In addition, to avoid possible learning effects, an extra CET was performed the week before the first training period. The patient was weighed on a calibrated medical scale (Kern, Model MPD 250K, Balingen-Frommern, Germany), wearing sportswear. Her height was measured using a Harpenden stadiometer (Holtain Ltd., Crosswell, Crymych, UK) to calculate BMI [mass (kg)/height (m^2^)]. Skinfold measurements were taken from the biceps, triceps, supra-illiac, and subscapular skinfold according to the published guidelines (42). Linear regression equations of Durnin and Womersley (43) were used to predict body density. Percentage of body fat was estimated using the equation by Siri (44).The patient was then familiarized with the exercise testing equipment and procedures.

#### Heart rate and heart rate variability measurements

An electrocardiographic recording system (AMEDTEC ECGpro^®^ CardioPart 12 USB, Aue, Germany) was used that meets specifications set by the American heart association (45). The ECG was obtained for 5 min while the patient rested quietly in a sitting position on the electronically braked cycle ergometer before each exercise test session. All measurements were taken at the same time of day (12:00 p.m., midday) in order to minimize circadian time effects. Prior to measurement, the patient rested comfortably for at least 15 min in a sitting position on a chair.

##### Signal processing

Heart rate time series consisting of successive beat-to-beat intervals (RR) were extracted from the raw data records. Afterwards, these time series were filtered by applying an adaptive variance estimation algorithm to remove and interpolate ventricular premature beats and artifacts (e.g., movement, electrode noise, and extraordinary peaks). The RR from the second up to the fourth minute was used for analysis. We obtained the baseline HR and the RMSSD as a time domain parameter of HRV. Non-linear properties of HR were investigated using the Poincaré plot. A return map was constructed by plotting each RR against the preceding RR. The variance of the resulting scattergram was characterized by the deviations SD1 and SD2. Here, we focused on the instantaneous variability reflected by SD1, which is related to parasympathetic activity (46). Because of a strong dependency of SD1 on basic heart rhythm we also estimated this index normalized by mean RR (SD1/RR_Mean_) (47).

#### Physical fitness assessment

The resting period was followed by a 3-min pedaling session at 6 W. Thereafter, CET was initiated from a baseline work rate of 15 W and a ramp slope of 15 W/min until the patient reached her limit of tolerance. Exhaustion occurred when the patient could not longer maintain the required power output. We verbally encouraged the patient to aim for a pedaling-frequency of 70–80 rpm and to give maximum effort until volitional exhaustion. HR and gas exchange measurements (MetaLyzer 3B; Cortex Biophysik, Leipzig, Germany; breath-by-breath system) were carried out throughout the test. The respiratory exchange ratio (RER) of carbon dioxide (CO_2_) output to oxygen (O_2_) uptake served as an objective control parameter for the degree of effort.

For data analysis, the breath-by-breath values were smoothed using a 15-breath moving average, aligned to the time of the central breath (48). Peak oxygen uptake (VO_2_peak), peak minute ventilation (VEpeak), and peak RER (RERpeak) were defined as the highest value of 15-breath average occurring during CET. The highest HR detected during CET was defined as HRpeak. The peak power output (Ppeak) was defined as the highest power output that was sustained for 1 min during the test. When the patient was not able to cycle to the end of the last 1-min interval, Ppeak was linearly interpolated based on the proportion of the time completed during the final stage.

For the description of aerobic capacity, the ventilatory threshold 1 (VT_1_), a common and reproducible submaximal indicator of endurance capacity, was chosen. VT_1_ was determined using a combined model including the following three methods: (i) the V-slope method (49): the first disproportionate increase in VCO_2_ determined from the VCO_2_–VO_2_ plot, (ii) the ventilatory equivalent method: an increase in VE/VO_2_ with no increase in VE/VCO_2_, and (iii) the excess carbon dioxide method: an increase from steady state to an excess production of CO_2_.

#### Training program

The patient trained twice a week (Tuesday and Friday) under supervision of an exercise physiologist. Each training session lasted 35 min including a standard warm-up (5 min) and cool-down (5 min). Training intensity was prescribed in relation to the power output corresponding to the VT_1_ and the VO_2_peak obtained in the respective preceding incremental exercise test. The warm-up and the cool-down periods were performed at power output of 70 and 40% of the VO_2_ at VT_1_, respectively. HR was continuously recorded throughout each training session using a HR monitor (RS800CX, Polar Electro, Kempele, Finland) and the Borg 6–20 scale was used to assess the rate of perceived exertion during each training session.

The moderate-intensity training (CT) consisted of 25 min of continuous exercise at a power output, which corresponds to 20% of the difference between the VT_1_ and VO_2_peak (20%), whereas the IT session consisted of 25 s × 30 s bursts at 80% with 30 s active recovery (a work to rest ratio of 1:1). During the 30-s recovery between bursts, the patient pedaled at a power output of 10 W. The exercise prescription of CT and IT was designed to apply different intensities. However, the same overall training load for each training session was achieved for the patient. The total amount of work (Watt × min) for the CT (62 W × 25 min) and IT [(111 W × 12.5 min) + (10 W × 12.5 min)] conditions were 1555 W × min and 1517 W × min, respectively. The resulting energy expenditure (EE) during CT (332 kcal) and IT (331 kcal) training sessions were comparable. The EE can be estimated using the American College of Sports Medicine’s metabolic calculation formula for leg cycling [VO_2_ = (10.8 × Watt × body mass^−1^) + 7]. The estimated training VO_2_ (l/min) can then converted into EE (kcal/min) by multiplying by 5 × total number of cycling minutes.

### Results

No significant change of PANNS rating was observed although the patient indicated some subjective improvements. Compliance of the patient with training requirements was excellent and without differences between CT and IT, although the IT was subjectively slightly more strenuous for the patient than the CT program (Borg: 16.8 ± 1.4 for the CT versus 18.3 ± 0.7 for the IT). Average HR during training was 141 ± 4 bpm in the CT (range 134–148 bpm) and 141 ± 5 bpm in the IT (131–149 bpm), respectively. The patient maintained almost constant body fat, whereas body mass went slightly down during the training period and increased after detraining (Table 1).

**Table 1:** **Anthropometric characteristics and maximal ergometric values of the patient before training, after 6-weeks continuous training, 6-weeks interval training, and a detraining of 6 weeks**.

	Baseline	Continuous training	Interval training	Detraining
Body mass (kg)	106.8	104.6	103.0	104.5
Body mass index (kg/m^2^)	40.7	39.9	39.2	39.8
Body fat (%)	46.6	46.1	46.3	46.7
HRpeak (beats/min)	146	153	152	154
Ppeak (W)	105	128	143	120
VO_2_peak (ml/kg/min)	15.7	17.0	19.1	17.2
VEpeak (l/min)	42.5	49.3	55.5	51.4
RERpeak	1.06	1.18	1.14	1.12

*HRpeak, peak heart rate; Ppeak, peak power output; VO_2_peak, peak oxygen uptake; VEpeak, peak minute ventilation; RERpeak, peak respiratory exchange ratio*.

#### Heart rate and heart rate variability

**Figure 2:**
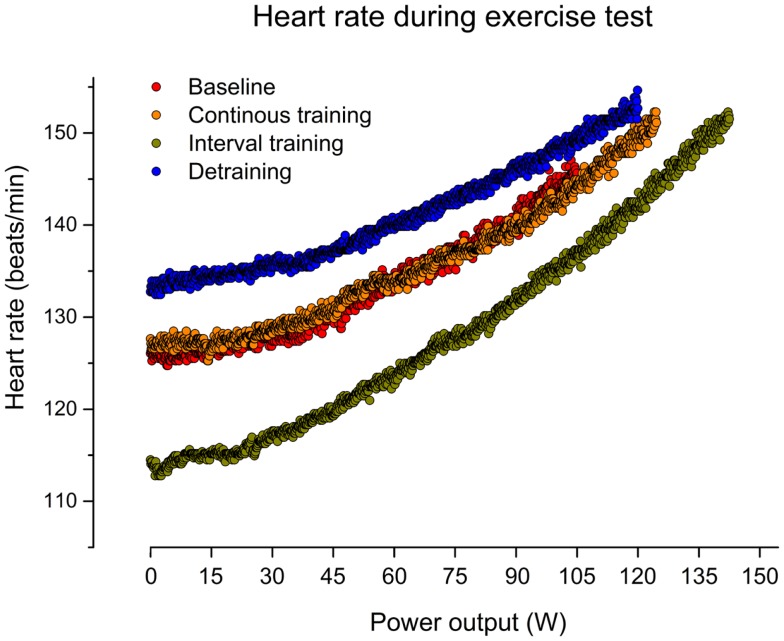
**Heart rate (beat-to-beat) during graded cardiopulmonary exercise test (CET) on a bicycle before training (baseline), after 6 weeks of continuous training, 6 weeks of interval training, and 6 weeks of detraining**.

As indicated in Figure 1, CT for 6 weeks did not affect the resting HR in our patient (mean HR: 121) in comparison to baseline (mean HR: 121). In contrast, IT for 6 weeks reduced the resting HR by about 14% (mean HR: 104). After discontinuation of training, the resting HR returned to the initial increased level (mean HR: 128). Figure 2 illustrates HRs during the CET at baseline, after CT, after IT, and after 6 weeks of detraining. Only the IT induced a pronounced effect on resting HR during exercise, which was lost after 6 weeks of detraining.

**Figure 3:**
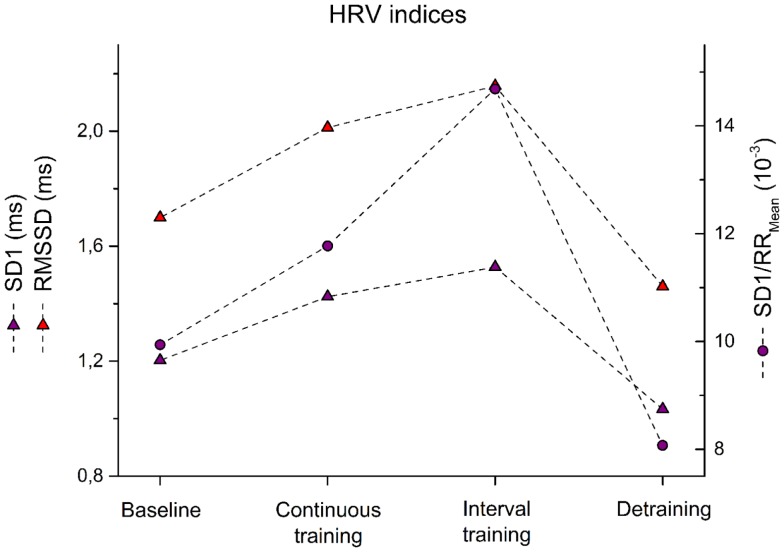
**Root mean of squared successive difference – RMSSD, instantaneous variability – SD1, and SD1 normalized by mean RR – SD1/RR_Mean_ are shown for the patient before training (baseline) after 6 weeks of continuous training, 6 weeks of interval training, and 6 weeks of detraining**.

Similarly, HRV indices reflected changes in autonomic regulation. IT was far more effective than moderate CT to increase HRV. Changes of HRV measures such as RMSSD, SD1, and SD1/RR_Mean_ are depicted in Figure 3. In particular, RMSSD showed an improvement to baseline after CT by 18% while IT led to 27% improvement. Similarly, SD1 increased after CT by 18 versus 27% after IT. Most pronounced was the effect when SD1 was adjusted to HR. Here, 18% after CT and 48% after IT were observed. All HRV measures returned to baseline levels within 6 weeks after detraining (Figure 3).

#### Changes of physical fitness

**Figure 4:**
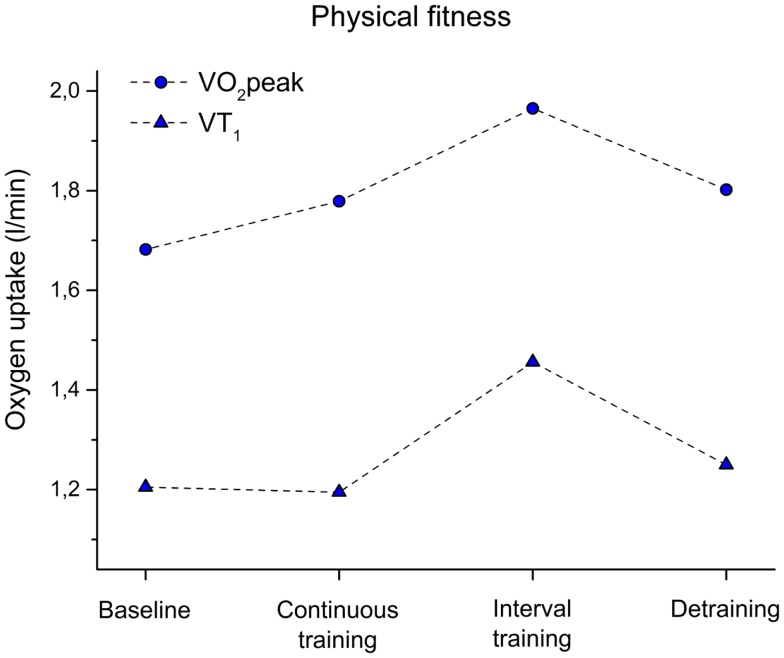
**Peak oxygen uptake (VO_2_peak) and oxygen uptake at the ventilatory threshold 1 (VT_1_) before training (baseline), after 6 weeks of continuous training, 6 weeks of interval training, and 6 weeks of detraining**.

As displayed in Figure 4, we did not observe a change of VT_1_ after CT when comparing baseline and fitness after training (−1%). Only a slight increase was observed as indicated by VO_2_peak (8% improvement) after CT. In contrast, after IT, a 21% increase was observed for VT_1_ and 22% for VO_2_peak.

## Discussion

Low cardiorespiratory fitness has been recognized as a strong and independent risk-factor for all-cause mortality in adults and a key risk-factor for coronary heart disease-related mortality (50–52). Reduced physical fitness has been reported in first-episode schizophrenia patients, as well as in more chronic patients (20, 53, 54). Research on exercise frequency and schizophrenia indicates that patients spend less time engaged in regular physical activity than the general population (55, 56). The health effects of regular physical activity are well established and include decreased mortality and morbidity related to cardiovascular disease and diabetes, as well as improvements in mental health, physical functioning, and body weight (57). However, the specific autonomic dysregulation in patients with schizophrenia might require particular physical interventions. Here, we present a case showing that a 6-week continuous exercise intervention neither altered cardiovascular regulation nor physical fitness. Although we have observed this in only one subject, we believe it is worth discussing to generate hypotheses for future interventional studies. Previous research suggested that continuous aerobic physical exercise is an effective method to increase physical fitness (i.e., peak oxygen uptake) and to decrease HR at rest by increasing its variability (HRV) (31). Moreover, previous studies by Rognmo et al. (21) and Helgerud et al. (40) emphasized the superiority of high-intensity training for VO_2max_ and VO_2peak_ in comparison to moderate-intensity exercise (21, 40). Similar effects were observed in our patient, including an increase of VO_2peak_ of 22% resulting from high-intensity training, in contrast to only 8% from CT. However, the study by Helgerud et al. (40) showed an influence of CT on HR while studying running economy in healthy subjects (40). No such effect of CT on HR was observed in our patient, even though she was completely sedentary before the exercise intervention started. The exercise physiologist who observed the training sessions and HR assessments confirmed that the correct amount of exercise was performed. In contrast, IT sessions, combining high-intensity bursts power output with low intensity recovery phases, revealed a significant effect both on cardiovascular regulation and physical fitness. It might indicate that patients with schizophrenia require a specific training program. It is well known that patients with schizophrenia suffer from a high internal stress level. It is likely that continuous exercise is an additional stressor for these patients, which might not yield the desired effect. However, IT consists of short bursts of physical strain followed by relaxation. Thus, the cardiovascular system of patients challenged in this fashion might respond more readily in comparison to continuous exercise for 30 min. Interestingly, yoga contains also an alternating stress–relaxation sequence. Duraiswamy et al. (58) showed that yoga had superior efficiency in comparison to exercise training (58). Similarly, a recent review of randomized control trials showed that yoga therapy might serve as a useful add-on treatment to reduce general psychopathology and positive and negative symptoms (59). Although yoga methods might differ enormously, it is thought to create inner, physical, and emotional balance through the use of body postures (called asanas) combined with breathing techniques (pranayama). This is of particular interest since breathing rates and rhythms are altered in the disease (17, 60). An alternative interpretation of our results could be that titration of exercise amount (volume and/or intensity) to elevated levels over a period of 12 weeks by changing from CT to IT increasing the amount halfway through produced beneficial effects. One might speculate that this was achieved by motivationally or otherwise enabling the subject to perform vigorous exercise after a 6-week run-in period of more moderate exercise. Future studies need to take this possibility into account to adequately calculate run-in periods and training durations for patients with schizophrenia.

In addition, it is important to realize how quickly HR and physical fitness improvement vanished after the intervention. Mean resting HR was in fact slightly increased in comparison to baseline (7 beats/min) after detraining. After such a long time of training, our patient was well accustomed to our laboratory and to the stuff. Thus, we can rather exclude major problems during adjustment. In addition, disease severity remained stable. Therefore, it is important to include post-training examinations in future studies to observe the stability of gained cardiovascular effects.

Our results are limited due to the presentation of a single case only. Additionally, the order of CT and IT might have influenced the better response to IT. Possibly, the 6 weeks CT training prepared the ground for the IT response. Therefore, randomized control trials are needed to investigate different exercise intervention schedules to understand physiological responses in these patients. Endurance training should be compared with IT or a combination of relaxation techniques and endurance training. This might help to change the physiological state. It is especially of great importance since psychiatrists tend to focus on changes of brain structure and function and thereby sometimes overlook physiological responses. However, we believe that physiological effects need to be included to estimate increased cardiovascular health in these patients. This is especially important since effects vanished so quickly.

## Concluding Remarks

Our case illustrates the need for comparing different exercise interventions in patients with schizophrenia, since cardiovascular dysfunction might interfere with training adaptations of the body normally observed. Although randomized control trials are mandatory for an evaluation of exercise interventions, our case illustrates that the physiological basis needs to be evaluated before effects of exercise on brain function and structure are analyzed in these patients.

## Conflict of Interest Statement

The authors declare that the research was conducted in the absence of any commercial or financial relationships that could be construed as a potential conflict of interest.
